# Screening of Botanical Drugs against SARS-CoV-2 Entry Reveals Novel Therapeutic Agents to Treat COVID-19

**DOI:** 10.3390/v14020353

**Published:** 2022-02-08

**Authors:** Junyuan Cao, Yang Liu, Minmin Zhou, Siqi Dong, Yuxia Hou, Xiaoying Jia, Xiaohao Lan, Yueli Zhang, Jiao Guo, Gengfu Xiao, Wei Wang

**Affiliations:** 1 State Key Laboratory of Virology, Center for Biosafety Mega-Science, Wuhan Institute of Virology, Chinese Academy of Sciences, Wuhan 430071, China; cjy444093101@live.com (J.C.); liuyang@wh.iov.cn (Y.L.); zhouminmin18@mails.ucas.ac.cn (M.Z.); dongsiqi19@mails.ucas.ac.cn (S.D.); houyuxia20@mails.ucas.ac.cn (Y.H.); jiaxiaoying17@mails.ucas.edu.cn (X.J.); 15092118139@163.com (X.L.); 2120191197@mail.nankai.edu.cn (Y.Z.); guojiao16@mails.ucas.ac.cn (J.G.); xiaogf@wh.iov.cn (G.X.); 2 University of the Chinese Academy of Sciences, Beijing 100049, China; 3 State Key Laboratory of Medicinal Chemical Biology, College of Pharmacy, Nankai University, Tianjin 300071, China

**Keywords:** SARS-CoV-2, entry inhibitor, angeloylgomisin O, schisandrin B, combination therapy

## Abstract

An escalating pandemic caused by the novel severe acute respiratory syndrome coronavirus 2 (SARS-CoV-2) has severely impacted global health. There is a severe lack of specific treatment options for diseases caused by SARS-CoV-2. In this study, we used a pseudotype virus (pv) containing the SARS-CoV-2 S glycoprotein to screen a botanical drug library containing 1037 botanical drugs to identify agents that prevent SARS-CoV-2 entry into the cell. Our study identified four hits, including angeloylgomisin O, schisandrin B, procyanidin, and oleanonic acid, as effective SARS-CoV-2 S pv entry inhibitors in the micromolar range. A mechanistic study revealed that these four agents inhibited SARS-CoV-2 S pv entry by blocking spike (S) protein-mediated membrane fusion. Furthermore, angeloylgomisin O and schisandrin B inhibited authentic SARS-CoV-2 with a high selective index (SI; 50% cytotoxic concentration/50% inhibition concentration). Our drug combination studies performed in cellular antiviral assays revealed that angeloylgomisin O has synergistic effects in combination with remdesivir, a drug widely used to treat SARS-CoV-2-mediated infections. We also showed that two hits could inhibit the newly emerged alpha (B.1.1.7) and beta (B.1.351) variants. Our findings collectively indicate that angeloylgomisin O and schisandrin B could inhibit SARS-CoV-2 efficiently, thereby making them potential therapeutic agents to treat the coronavirus disease of 2019.

## 1. Introduction

The coronavirus disease of 2019 (COVID-19), caused by severe acute respiratory syndrome coronavirus-2 (SARS-CoV-2), poses a severe threat to global public health, affecting economic and social stability, and requires the rapid development of novel treatment and preventive measures [1]. The coronavirus is an enveloped virus with four structural proteins: spike (S), membrane (M), envelope (E), and nucleocapsid (N) proteins. SARS-CoV-2 shares a high degree of sequence identity with the previously emerged SARS-CoV and exploits the same human cell receptor, angiotensin-converting enzyme 2 (ACE2), for infection [2,3]. The mechanism by which the receptor-binding domain (RBD) in the S1 subunit of the S protein on the virion binds to the ACE2 receptor on the target cell has been elucidated. Moreover, the heptad repeat 1 (HR1) and 2 (HR2) domains in the S2 subunit of the S protein interact with each other to form a six-helix bundle fusion nucleus. This interaction brings the virus membrane and cell membrane close together to facilitate fusion and infection [4,5].

It is imperative to carry out SARS-CoV-2 cultures and assays in a biosafety level (BSL)-3 laboratory. The vesicular stomatitis virus (VSV) reverse genetics system is a high-throughput assay that can provide a safe, reliable, and stable platform to study SARS-CoV-2 S-glycoprotein inhibition by antibodies or small molecules. Researchers have also employed related viral systems that afford virus entry studies in BSL-2 laboratories to facilitate rapid inhibitor screening using fluorescence or luminescence-based reporters [6]. 

More than a year after its emergence, SARS-CoV-2 continues to cause a fervor, with the identification of new variants from the United Kingdom, South Africa, Brazil, and India [7,8,9]. The G614 variant was first detected in early 2020 and contains a D614G substitution in the RBD of its S protein, which appears to increase viral transmissibility [10,11,12]. As other new variants have emerged over the past few months, the primary focus of research has been to identify further RBD substitutions and their impacts on viral infection. The recent emergence of SARS-CoV-2 variants of concern, such as alpha (B.1.1.7), was first identified in the United Kingdom, beta (B.1.351) in South Africa, gamma (P.1) in Brazil, and delta (B.1.617.1) in India. S-mediated virus entry is the first step of SARS-CoV-2 infection. These variants of concern were defined by eight to ten amino acid substitutions or deletions in the S protein, which has raised concerns about increased virus transmissibility [13,14,15]. 

Natural compounds have been universally used to prevent and cure various illnesses in many countries since ancient times. The isolation and characterization of the structures of selected natural products have become critical contributors to pharmaceuticals, with several critical pharmaceuticals being modeled after natural products [16,17].

This study focuses on screening drugs targeting the entry step of SARS-CoV-2 infection to block the early stages of viral infection and spread. Using the SARS-CoV-2 S VSV-based-pseudovirus (SARS-CoV-2 S pv), we identified that angeloylgomisin O, schisandrin B, procyanidin, and oleanonic acid could successfully inhibit SARS-CoV-2 entry by inhibiting membrane fusion. Our findings potentially offer new therapeutic strategies for the treatment of COVID-19.

## 2. Materials and Methods

### 2.1. Cells Lines 

HEK 293T, Vero E6, Caco-2, and BHK-21 cells were obtained from the American Type Culture collection (ATCC, Manassas, VA, USA). HEK 293T, Vero E6, Caco-2, and BHK-21 cells were maintained at 37 °C, 5% CO_2_, in Dulbecco’s modified Eagle’s medium (DMEM; Gibco), supplemented with 10% fetal bovine serum (FBS, Gibco).

### 2.2. Viruses

The pseudotype VSV bearing the S protein of SARS-CoV-2 S (GenBank QHD43416.1) and MERS-CoV S (GenBank NC_019843.3) were generated as previously reported [18,19]. The 293T cells transfected with pcDNA3.1-SARS-CoV-2 S (ct19), pcDNA3.1-SARS-CoV-2 S (wt), or pcDNA3.1-MERS-CoV S (ct16) for 48 h were infected with pseudotype VSV (described below), in which the G gene was replaced with the luciferase gene at an MOI of 0.1 for 2 h. The culture supernatants were harvested 24 h later, centrifuged to remove cell debris, and stored at −80 °C. To generate VSV-G pseudotype VSV, BHK-21 cells in 6-well plates were infected with a recombinant vaccinia virus (vTF7-3) encoding T7 RNA polymerase at an MOI of 5. After 45 min, the cells were transfected with 11 μg of mixed plasmids with a 5:3:5:8:1 ratio of pVSVΔG-eGFP-GPC (pVSVΔG-Rluc to generate pseudotype VSV), pBS-N, pBS-P, pBS-G, and pBS-L. After 48 h, the supernatants were filtered to remove the vaccinia virus and inoculated into BHK-21 cells that had been transfected with pCAGGS-VSV G 24 h previously. The titer of the pseudotype virus was measured by infecting BHK-21 cells previously transfected with pCAGGS-VSV G and determined by plaque assay 24 h post-infection (h.p.i.). The titer of SARS-CoV-2 S pv (ct19) was 1.5 × 10^7^ PFU/mL. Point mutants were made from pcDNA3.1-SARS-CoV-2 S (ct19) by using a fast mutagenesis system (Transgene Biotech, Bordeaux, France), and mutations were confirmed by sequencing (Sangon, Shanghai, China). We used the same method to generate the SARS-CoV-2 S variants pv.

The SARS-CoV-2 isolate WIV04 (GISAID accession number EPI_ISL_402124) was isolated from Huh7 cells from the original sample and was passaged in Caco-2 cells. The viral titer used the plaque assay determined in Vero E6 cells. The alpha (B.1.1.7, IVCAS6.7552) and beta (B.1.351, NPRC 2.062100001, GenBank: MW789246.1) variants were provided by the National Virus Resource Center.

### 2.3. Materials

Schisandrin B (CAS No. 61281-37-6), procyanidin (CAS No. 20347-71-1), and oleanonic acid (CAS No. 17990-42-0) were purchased from Selleck (Houston, TX, USA) and Weikeqi Biotech (Sichuan, China). Angeloylgomisin O (CAS No. 83864-69-1) was purchased from MCE (Sherman Oaks, CA, USA) and Weikeqi Biotech (Chengdu, China). 25-Hydroxycholesterol, Camostat, and Remdesivir were purchased from MCE (Sherman Oaks, CA, USA). All compounds were dissolved in DMSO for subsequent experiments.

### 2.4. Viral Copies Assay

Equal volumes of SARS-CoV-2 S pv (ct19) and SARS-CoV-2 S pv (wt) were treated with DNase and RNase. RNA from the viruses was extracted using Trizol (TaKaRa) and reverse transcribed using the PrimeScript™ RT reagent Kit (TaKaRa). Viral particles were quantified via RT-qPCR using a specific primer pair to detect VSVΔG-Rluc (primers 5′-GTAACGGACGAATGTCTCATAA-3′ and 5′-TTTGACTCTCGCCTGATTGTAC-3′).

### 2.5. Immunoblotting

Equal volumes of virus particles were precipitated with acetone and lysed using RIPA lysis buffer. Lysates were treated with loading buffer, subjected to SDS-PAGE, and then transferred onto a polyvinylidene difluoride (PVDF) membrane (Millipore). SARS-CoV-2 S was detected using a rabbit anti-S2 subunit mouse monoclonal antibody (GeneTex, GTX632604;1:2000 dilution). Horseradish peroxidase-linked goat anti-mouse IgG antibody (Proteintech, Chicago, IL, USA, 1:5000) was used.

### 2.6. HTS Optimization and Assaying of the Botanical Drug Library

A library of 1037 botanical compounds was purchased from Weikeqi Biotech (Sichuan, China). The compounds were collected and stored in 10-mM stock solutions in DMSO at −80 °C until use. The first round of HTS was carried out, and Caco-2 cells were seeded at a density of 2.4 × 10^4^ cells per well in 96-well plates. After incubation overnight, cells were treated in duplicate with the compounds (50 μM), and 1 h later, cells were infected with SARS-CoV-2 S pv (ct19) with the MOI = 5, and the supernatant was removed 1 h.p.i. Camostat 100 μM and 0.5% DMSO were used as positive and negative controls, respectively. After 24 h, the luciferase activity was measured using the Rluc assay system (Promega, Madison, WI, USA). The primary compounds were then secondarily screened using VSV pv (MOI of 0.5) to rule out VSV genome replication inhibitors and Rluc activity. 

### 2.7. Cell Viability

Caco-2 cells were seeded at a density of 2.4 × 10^4^ cells per well in 96-well plates. After incubation overnight, cells were treated in duplicate with the compounds for 24 h. The cell supernatant was removed, and 50-μL 0.5% 3-(4,5-dimethyl-2-thiazolyl)-2,5-diphenyl-2H-tetrazolium bromide (MTT; Sigma-Aldrich) dissolved in PBS were added to the cells. In actively growing cells, MTT can utilize reduced nicotinamide adenine dinucleotide (NADH)- and reduced nicotinamide adenine dinucleotide phosphate (NADPH)-dependent cellular oxidoreductase enzymes, producing a blue formazan product that is freely soluble in DMSO. After incubation at 37 °C for 4 h, the supernatant was removed carefully, and 50-μL DMSO was added to the cells. After being gently shaken, the plates were measured at 492 nm using a spectrophotometer, and cell viability was calculated.

### 2.8. SARS-CoV-2 Binding Assay

The binding reaction was performed using the SARS-CoV-2 inhibitor screening ELISA kit (Sino Biological, Beijing, China). Four hits were incubated with hACE-his and then transferred into 96-well plates coated with SARS-CoV-2 RBD. One hour later, the plates were washed with the buffer and incubated with HRP-labeled anti-his antibody. After incubation for another 1 h at room temperature, the plates were washed and detected with the color regent substrate. Finally, the reaction was stopped with 1-M H2SO4, and the optical density (OD) at 450 nm was measured [20,21]. In a binding assay, the SARS-CoV-2 spike antibody (40150-D002, Sino Biological, Beijing, China) was used as the positive control.

### 2.9. Virucidal Assay

To study the virucidal effects, SARS-CoV-2 (10^6^ PFU/mL) was incubated with hits (25 μM and 0.25 μM) or the vehicle at 37 °C for 1 h. The samples were diluted in DMEM and calculated the remaining viral titer using the plaque assay. Viral titers were determined at dilutions that the hits were not effective (more than 100× dilution).

### 2.10. Membrane Fusion Assay

Vero E6 cells were co-transfected with pcDNA3.1-SARS-CoV-2 S (ct19) (0.25 μg) and pEGFP-N1 (0.25 μg) by using lipo2000 in 24-well plates. After transfection for 4 h, the medium was replaced with 2% DMEM containing different concentrations of the hits. After 24 h, syncytium formation was visualized using an M-shot image system (Micro-shot Technology, China). A dual split protein (DSP)-based cell–cell fusion assay was used to detect SARS-CoV-2 S (ct19) inhibitory activity of the four hits [22]. Briefly, a total of 3 × 10^4^ 293T cells (effector cells) were seeded in a 96-well plate, and 3 × 10^5^ cells/mL 293T (target cells) were seeded in a 6-well plate culture, and then, the cells were incubated at 37 °C. On the next day, the effector cells were co-transfected with SARS-CoV-2 S (ct19) and a DSP1-7 plasmid, the target cells were transfected with DSP8-11 plasmid, and then, the cells were incubated at 37 ℃. After 24 h, serially diluted four hits were added to the effector cells were incubated for 1 h; the target cells were resuspended at 3 × 10^5^ cells/mL in a prewarmed culture medium that contained EnduRen live cell substrate (Promega) and hits. Then, target cells were transferred to the effector cells, and a mixture of cells was spun down to maximize cell–cell contract. After incubation for 6 h, the luciferase activity was measured. In the DSP-based cell–cell fusion assay, clofazimine (10 μM) was used as the positive control.

### 2.11. Antiviral Effect of Hits against SARS-CoV-2 and SARS-CoV-2 Variants of Concern

Caco-2 cells were seeded at a density of 2.4 × 10^4^ cells per well in 96-well plates. After overnight incubation, cell monolayers were treated with hits; 1 h later, cells were infected with SARS-CoV-2 or SARS-CoV-2 variants at an MOI of 0.5. After an additional 24 h of incubation, the infection was stopped by rinsing each well, and the cells were fixed with 4% paraformaldehyde. Ten micrometers remdesivir was used as the positive control.

### 2.12. Immunofluorescence Assay (IFA)

Cells were permeabilized using PBS with 0.2% Triton X-100 for 15 min and blocked with 5% FBS (Gibco), followed by treatment with the primary antibody anti-SARS-CoV-2 NP (GeneTex GTX635678, Irvine, CA, USA) at a 1:500 dilution overnight at 4 °C. After six rinses with PBS, the cells were stained with DyLight 488-labeled antibody against rabbit IgG (KPL, Gaithersburg, MD, USA). Nuclei were stained with 4′,6-diamidino-2-phenylindole (DAPI, Sigma-Aldrich, St. Louis, MO, USA). Nine fields per well were imaged, and the percentages of infected and DAPI-positive cells were calculated using Harmony 3.5 software.

### 2.13. Drug–Drug Interactions of Remdesivir with Hits

Caco-2 cells were seeded at a density of 2.4 × 10^4^ cells per well in 96-well plates. After overnight incubation, different final concentrations of remdesivir were added to each row of the 96-well plate. Simultaneously, final concentrations of the four hits (50, 25, 12.5, 6.25, 3.125, 1.5625, 0.78125, and 0 μM) and remdesivir (20, 4, 0.8, 0.16, and 0 μM) were added to each column of the plate. The cells were infected with SARS-CoV-2 at an MOI of 0.5. After an additional 24 h of incubation, the infection was stopped by rinsing each well, and the cells were fixed with 4% paraformaldehyde. The antiviral activities were determined using the IFA assay. To determine the drug–drug interactions, the results were analyzed by MacSynergy II using the manual. Differential surface plots at the 95% confidence level (CI) were generated according to the Bliss independence model. The volumes of the synergistic regions were equal to the relative quantity of synergy produced per change in the two drugs concentrations.

## 3. Results

### 3.1. Construction of SARS-CoV-2 S pv (ct19)

Firstly, we constructed a SARS-CoV-2 S pv based on a VSV backbone to perform high-throughput screening under BSL-2 conditions. The cytoplasmic tail of the S glycoproteins of SARS-CoV-2 is highly similar to that of SARS-CoV and carries signals for their retention in the endoplasmic reticulum. Previous studies have found that 19 amino acid deletions in the cytoplasmic tail of SARS-CoV or SARS-CoV-2 S glycoprotein increased the infectivity of the single-cycle pseudotype virus [23,24,25,26].

For this study, we generated two recombinant plasmids expressing either the wild-type (wt) S glycoprotein, pcDNA3.1-SARS-CoV-2 S, or the truncated S glycoprotein with a deletion of 19 amino acids from its C-terminus, pcDNA3.1-SARS-CoV-2 S (ct19). These plasmids were used to generate SARS-CoV-2 S pv (wt) or the shortened S glycoprotein, SARS-CoV-2 S pv (ct19), respectively.

**Figure 1:**
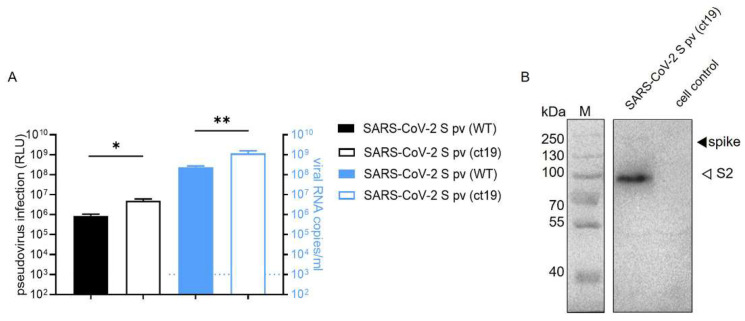
Construction of SARS-CoV-2 S pv (ct19). (**A**) The left axis shows the RLU (relative light unit) value detected 24 h after pseudovirus SARS-CoV-2 S pv (ct19) and SARS-CoV-2 S pv (wt) infection. The right *y*-axis shows virus copy number assays of both viral particles. (**B**) Immunoblots verifying the incorporation of the SARS-CoV-2 spike protein in the pseudovirus. The cell control was the culture supernatant of 293T cell transfected with pcDNA3.1 48 h and infected with VSV pv. Data are presented as the means ± standard deviations (SDs) for more than 2 independent ex-pediments (* *p* < 0.05, ** *p* < 0.01).

As shown in Figure 1A, the Renilla luciferase (Rluc) activity of the cells infected with SARS-CoV-2 S pv (ct19) was higher than that of the cells infected with SARS-CoV-2 S pv (wt). Next, the VSV copy number was assayed in the different pv harvests, and a higher packaging efficiency was detected in the SARS-CoV-2 S pv (ct19) system than in SARS-CoV-2 S pv (wt) (Figure 1A), reaching 1.15 × 10^9^ copies/mL and 2.3 × 10^8^ copies/mL, respectively. Immunoblotting confirmed the expression and accumulation of the SARS-CoV-2 S protein in SARS-CoV-2 S pv (ct19) (Figure 1B), enabling us to use SARS-CoV-2 S pv (ct19) for high-throughput screening (HTS) of the potential botanical drug candidates.

### 3.2. SARS-CoV-2 S pv (ct19) Entry Inhibitor Screening

**Figure 2:**
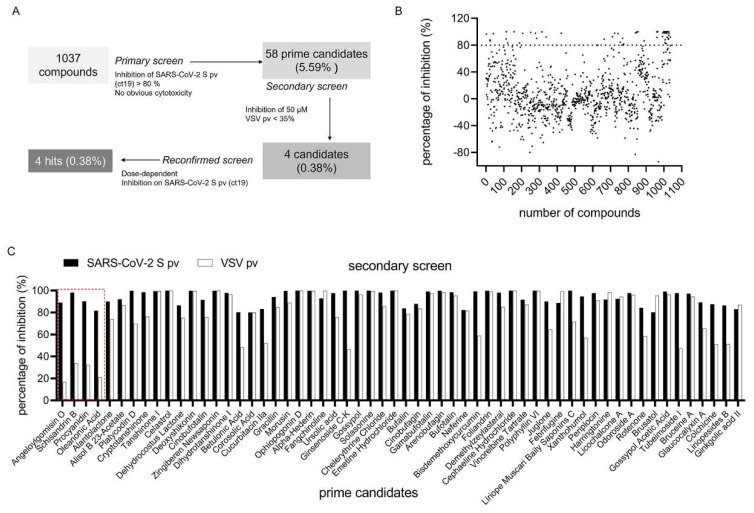
HTS for inhibitors of SARS-CoV-2 entry from a botanical drug library. (**A**) HTS assay flowchart. (**B**) HTS of a library of 1037 natural extracts for primary candidates inhibiting SARS-CoV-2 S pv (ct19) infection. Each dot represents the percent inhibition achieved by each compound at a concentration of 50 μM. (**C**) Inhibition of 58 candidates against SARS-CoV-2 S pv entry > 80%. The second screen of the 58 selected compounds. VSV pv was used as a control to exclude compounds targeting the backbone. The hits depicted in red box showed mild inhibition of VSV pv infection and were designated hits.

The HTS assay conditions were optimized to a seeding density of 2.4 × 10^4^ for Caco-2 cells and an MOI of 5 for the SARS-CoV-2 S pv (ct19) infective dose per well in 96-well plates. Under these optimized conditions, the signal/background (S/B) ratio, coefficient of variation, and Z’ factor were 1286.09, 7.35%, and 0.705, respectively, making this assay promising for large-scale inhibitor screening. As shown in Figure 2A, the HTS assay screened a library containing 1037 botanical drugs. Compounds with >80% inhibition and no apparent cytotoxicity at a concentration of 50 μM were defined as prime candidates, and 58 (5.59%) prime candidates (Figure 2A,B) were selected for further investigations. These prime candidates were then counter-screened to rule out the inhibition of VSV genome replication and Rluc activity. Four candidates (0.38%): angeloylgomisin O, schisandrin B, procyanidin, and oleanonic acid passed the secondary screen with a mild inhibition of VSV (16.85%, 33.65%, 21.02%, and 32.41%, respectively) at 50 μM (Figure 2C). This result indicated that these four hits specifically inhibited SARS-CoV-2 S infection at the entry step.

### 3.3. Four Hits Specifically Inhibited SARS-CoV-2 S-Mediated Entry by Dose-Dependent Inhibition

**Figure 3:**
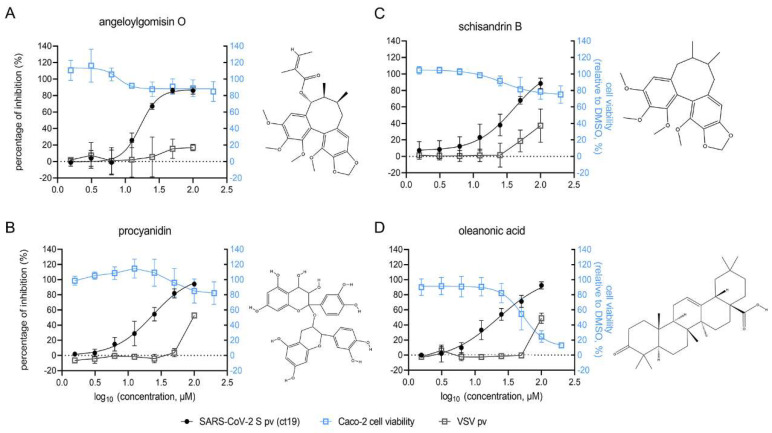
Dose-response curves and the structures of the four hits: angeloylgomisin O (**A**), schisandrin B (**B**), procyanidin (**C**), and oleanonic acid (**D**) for inhibiting SARS-CoV-2 S pv (ct19) infection. (**Right**) The structure of each hit. Caco-2 cells were seeded at a density of 2.4 × 10^4^ cells per well in 96-well plates. After overnight incubation, cell monolayers were treated with hits; 1 h later, cells were infected with different pv, and the supernatant was removed 1 h post-infection. The infected cells were lysed 23 h later, and the luciferase activities were measured. Cell viability was evaluated using the MTT assay. Hits at the indicated concentrations were added to pre-seeded Caco-2 cells in 96-well plates. Twenty-four hours later, cell viability was measured. Data are represented as the mean ± standard deviation (SD) from three to four experiments.

The dose-dependent inhibition of the four hits was further investigated using Caco-2 cells. As shown in Figure 3, all four hits suppressed SARS-CoV-2 S pv (ct19) infection in a dose-dependent manner from 100 μM to 1.5625 μM. Angeloylgomisin O inhibited SARS-CoV-2 S pv (ct19) entry by 90% at 100 μM and had little effect on VSV pv, demonstrating its specificity against SARS-CoV-2 (Figure 3A). Meanwhile, the cell viability was >80% when the inhibitors were used at an extremely high concentration (400 μM). Schisandrin B inhibited the entry of SARS-CoV-2 S pv (ct19) by 90% at 100 μM, whereas the cell viability was approximately 80% at 400 μM (Figure 3B). At 50 μM and 100 μM, procyanidin inhibited SARS-CoV-2 S pv (ct19) by approximately 80% and 95%, respectively, while the cell viability was approximately 80% at 400 μM (Figure 3C). Oleanolic acid (50 μM) inhibited SARS-CoV-2 S pv (ct19) entry by approximately 70% (Figure 3D). It was found to be relatively toxic to Caco-2 cells, with a 50% cytotoxic concentration (CC50) of 54 μM. The four hits were also purchased from other commercial sources and tested to validate their antiviral effects. All the compounds showed similar antiviral effects, conforming the inhibitory effect of the four hits.

### 3.4. Effects of Four Hits on Different Stages of SARS-CoV-2 Entry

**Figure 4:**
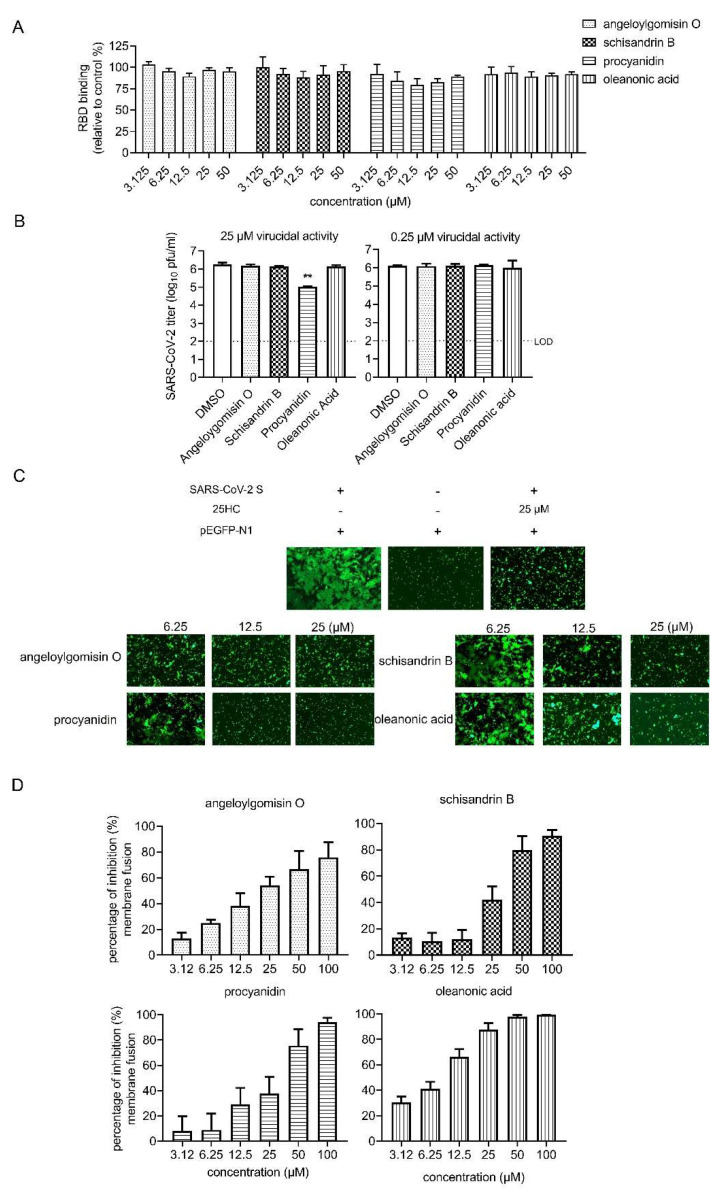
Effects of four hits on different stages of SARS-CoV-2 entry. (**A**) Effect of four hits on the binding between ACE2 and SARS-CoV-2 RBD. SARS-CoV-2 RBD was coated on the 96-well plates, followed by incubation with hACE-his in the different concentrations of the four hits or vehicle (DMSO) for 1 h. After washing with PBS, the HRP-conjugated anti-his antibody was incubated with the plates. After adding a color reagent for 15 min, the OD450 was measured, and the efficiency of RBD binding was normalized with the vehicle control. (**B**) Result of the virucidal assay. (**Left**) SARS-CoV-2 was incubated with DMSO or the hits (25 μM for 1 h). The treated virus was determined by the plaque assay. (Right) SARS-CoV-2 was incubated with DMSO or the hits (0.25 μM for 1 h). (**C**) SARS-CoV-2 S (ct19)-mediated cell–cell fusion on Vero E6 cells. (Left) Vero E6 cells that were co-transfected with SARS-CoV-2 S (ct19) and GFP plasmid. (**Middle**) Cells were transfected with GFP. (**Right**) Cells were co-transfected with SARS-CoV-2 S (ct19) and GFP and treated with 25-μM 25HC. Four hits inhibited SARS-CoV-2 S (ct19)-mediated membrane fusion in a dose-dependent manner. (**Bottom**) Vero E6 cells were co-transfected with SARS-CoV-2 S (ct19) and GFP later treated with the four hits in different concentrations. Syncytium formation was visualized 24–36 h later using fluorescent microcopy. Images are representative fields from three independent experiments. (**D**). Inhibition of SARS-CoV-2 S (ct19) protein-mediated cell–cell fusion by the inhibitors, determined by a DSP-based cell fusion assay. The effector cells were co-transfected with SARS-CoV-2 S (ct19) and a DSP1-7 plasmid, and the target cells were transfected with DSP8-11 plasmid. The cell fusion activity was quantitatively determined by measuring the luciferase activity. Data are presented as the means ± standard deviations (SDs) for more than 2 independent experiments (** *p* < 0.01).

A series of entry events were examined to dissect which step was blocked by the hits. Firstly, the effect of the inhibition on the receptor binding was tested, and as shown in Figure 4A, none of the hits had an effect on RBD binding. Next, we tested whether four hits could interact with SARS-CoV-2 and exert a virucidal effect. To this end, SARS-CoV-2 was mixed with hits at 25 μM or 0.25 μM for 1 h, and we determined the remaining viral titer using the plaque assay. SARS-CoV-2 infectivity was not reduced by incubation with angeloylgomisin O, schisandrin B, and oleanonic acid. While a reduction of >90% (>1 log10) was observed in the 25-μM procyanidin group, indicating that procyanidin exhibited a virucidal effect on the SARS-CoV-2 authentic virus. Previous evidence has shown that procyanidins can interact with synthetic membranes and protect them from oxidation and disruption [27]. Procyanidins might interact with the viral membrane and result in low infectivity. 

To further confirm the hit mechanisms, the effects of the four hits on SARS-CoV-2 S-mediated membrane fusion were examined. Recent research has identified that SARS-CoV-2 induces syncytia formation in the lungs of patients with COVID-19 [28]. It has been reported that cells infected with SARS-CoV-2 exhibit a typical syncytium phenomenon [5]. In this assay, fusion activity was conducted by co-transfecting SARS-CoV-2 S (ct19) and green fluorescent protein (GFP) into Vero E6 cells. After 24 h, the S protein of SARS-CoV-2 induced cell–cell fusion, resulting in the formation of syncytia (Figure 4C). 25-Hydrocholesterol (25HC) was used as the positive control for this assay [29,30]. As shown in Figure 4C, all four hits induced a dose-dependent reduction in syncytium size. Angeloylgomisin O blocked syncytia formation starting at a concentration of 6.25 μM, while procyanidin, oleanonic acid, and schisandrin B required higher concentrations (12.5 or 25 μM) to achieve the same (Figure 4C). To further quantitatively evaluate the inhibition, fusion efficacy was determined using the dual split protein assay. As shown in Figure 4D, four hits exhibited a dose-dependent inhibition of SARS-CoV-2 S-mediated membrane fusion. These results indicate that angeloylgomisin O, schisandrin B, procyanidin, and oleanonic acid could inhibit SARS-CoV-2 entry by inhibiting membrane fusion.

### 3.5. Four Botanical Hits Inhibited Authentic SARS-CoV-2 Infection

**Figure 5:**
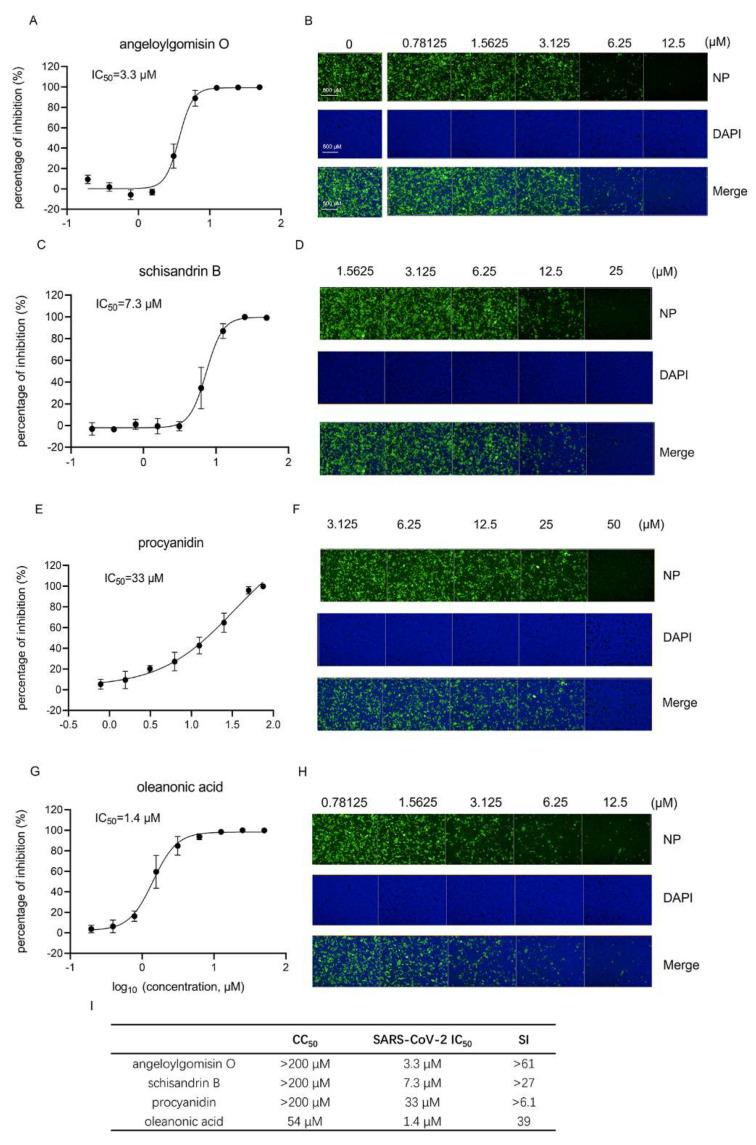
The four hits inhibit authentic SARS-CoV-2 infection. IFA analysis and images of angeloylgomisin O (**A**,**B**), schisandrin B (**C**,**D**), procyanidin (**E**,**F**), and oleanonic acid (**G**,**H**) inhibition of SARS-CoV-2 infection in Caco-2 cells. Caco-2 cells were seeded in 96-well plates. After overnight incubation, cell monolayers were treated with hits; 1 h later, the cells were infected with SARS-CoV-2 at an MOI of 0.5, followed by incubation for 24 h. IFA images showing the viral NP (green) and cell nuclei (blue) are displayed for Caco-2 cells. Cells were treated with different concentrations of angeloylgomisin O (**B**), schisandrin B (**D**), procyanidin (**F**), and oleanonic acid (**H**). (**I**) CC_50_, IC_50_, and SI values of four hits. Data were presented as the means ± SD from three independent experiments.

Next, an immunofluorescence assay (IFA) was performed using an anti-SARS-CoV-2 NP antibody to evaluate the inhibition of four hits on authentic SARS-CoV-2. As shown in Figure 5, all four hits inhibited authentic SARS-CoV-2 in a dose-dependent manner. The 50% inhibition concentration values (IC_50_) of angeloylgomisin O (3.7 μM), schisandrin B (7.3 μM), and oleanonic acid (1.4 μM) were less than 10 μM, while the IC_50_ value of procyanidin was 33 μM. It seemed that angeloylgomisin O, schisandrin B, and oleanonic acid exerted a more potent inhibition of authentic SARS-CoV-2.

The selective index (SI; CC_50_/IC_50_) was calculated using an authentic SARS-CoV-2 inhibition assay. The SI values of angeloylgomisin O and schisandrin were >61 and >27, respectively. This observation suggests that both drugs are promising candidates for COVID-19 treatment.

### 3.6. Effects of the Four Hits against MERS-CoV-S pv and SARS-CoV-2 S Variants pv

**Figure 6:**
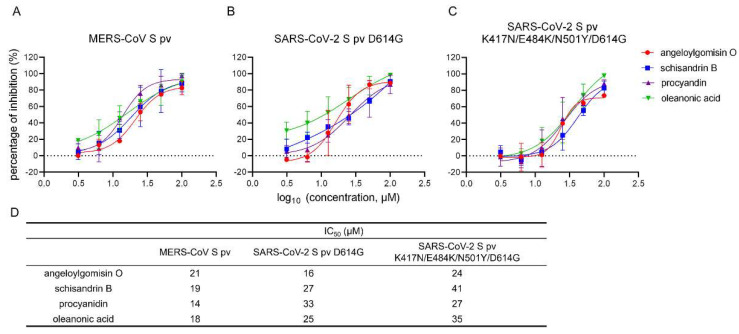
Antiviral activity of the four hits against MERS-CoV S pv, the SARS-CoV-2 S pv D614G mutant, and the SARS-CoV-2 S pv K417N/E484K/N501Y/D614G mutant. (**A**–**C**) Dose-dependent curves of both the four hits against MERS-CoV-S pv (**A**), SARS-CoV-2 S pv D614G (**B**), and the SARS-CoV-2 S pv K417N/E484K/N501Y/D614G mutant. (**D**) The IC_50_ of the four hits for inhibition against the MERS-CoV-S pv and variant pv. Caco-2 cells were seeded at a density of 2.4 × 10^4^ cells per well in 96-well plates. After overnight incubation, the cells were treated with the hits; 1 h later, the cells were infected with MERS-CoV-S pv, SARS-CoV-2 S pv D614G, and SARS-CoV-2 S pv K417N/E484K/N501Y/D614G. The infected cells were lysed 23 h later, and the luciferase activities were measured. Data were presented as the means ± SD from three independent experiments.

To verify the antiviral effects of the four hits on MERS-CoV S pv and SARS-CoV-2 S pv variants containing S protein mutations, we verified the inhibitory effects of the four hits against other coronaviruses and the SARS-CoV-2 variants. As shown in Figure 6A, all four hits inhibited the entry of MERS-CoV S pv into Caco-2 cells in a dose-dependent manner. The IC_50_ values of angeloylgomisin O, schisandrin B, procyanidin, and oleanonic acid against MERS-CoV S pv were 21 μM, 19 μM, 14 μM, and 18 μM, respectively.

A SARS-CoV-2 S pv variant containing the S protein D614G substitution was constructed and used to evaluate the inhibitory effect of the selected drugs. The G614 variant appears to increase the viral transmissibility [10,11,12]. The G614 variants have become dominant in the currently circulating virus strains. The variants of concern include the D614G substitution. As shown in Figure 6B, all four hits suppressed the entry of SARS-CoV-2 S pv D614G into Caco-2 cells in a dose-dependent manner. The IC_50_ values of angeloylgomisin O, schisandrin B, procyanidin, and oleanonic acid, at which they inhibited the entry of SARS-CoV-2 S pv D614G, were 16 μM, 27 μM, 33 μM, and 25 μM. We also introduced three additional amino acid substitutions, K417N, E484K, and N501Y, in the S D614G protein for our next set of investigations. These mutations have been observed in the beta variant and play an essential role in immune escape [13,14,15]. The results showed that all four hits inhibited SARS-CoV-2 S pv K417N/E484K/N501Y/D614G in a dose-dependent manner (Figure 6C). The IC_50_ values of angeloylgomisin O, schisandrin B, procyanidin, and oleanonic acid against SARS-CoV-2 S pv K417N/E484K/N501Y/D614G were 24 μM, 41 μM, 27 μM, and 35 μM, respectively, indicating the potential of all the four hits to inhibit new variants. 

### 3.7. Combinatory Treatments with the Drug Pair Remdesivir–Angeloylgomisin O Showed Enhanced Antiviral Activity

As all four hits could inhibit S-mediated membrane fusion, we assessed the efficacy of the combined treatment using remdesivir, a SARS-CoV-2 viral RNA-dependent RNA polymerase inhibitor [31,32], and the four hits. According to the bliss independence model, the degree of interaction was determined using MacSynergy II software to analyze the results [33,34]. The volumes of statistically significant synergy were evaluated, and a combination volume (CV) of >25 μM^2^ % was interpreted as evidence of synergy and >100 μM^2^ % as strong synergy [35].

**Figure 7:**
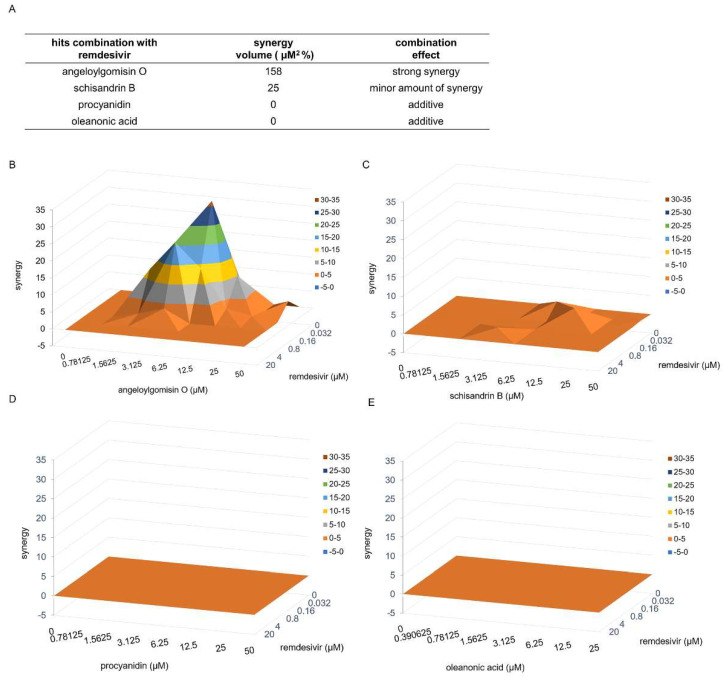
Combinatory treatments with the drug pairs remdesivir-angeloylgomisin O and remdesivir–schisandrin B show enhanced antiviral activity. (**A**) Synergy volumes for pairwise combination studies performed between remdesivir and each hit. Three-dimensional plot of synergy and antagonism at 99% confidence for the pairwise combination of remdesivir with angeloylgomisin O (**B**), schisandrin B (**C**), procyanidin (**D**), and oleanonic acid (**E**). The analysis was performed with MacSynergy II software.

Our results showed strong synergy interactions between remdesivir and angeloylgomisin O. Combination studies with remdesivir and angeloylgomisin O led to a CV of 158 μM^2^ % (Figure 7A). For combinations of remdesivir–angeloylgomisin O, the maximal synergistic effect was seen at a concentration of 0.032–0.8 μM remdesivir and 1.56–6.25 μM angeloylgomisin O. Remdesivir and schisandrin B showed a minor amount of synergy (CV was 25 μM^2^ %) (Figure 7A,C). Moreover, two drug combinations, procyanidin–remdesivir and oleanonic acid–remdesivir, had an additive antiviral inhibition profile (Figure 7D,E). 

### 3.8. Angeloylgomisin O and Schisandrin B Inhibited SARS-CoV-2 S Variants

Next, the usefulness of angeloylgomisin O and schisandrin B needs to be investigated in the context of the currently circulating SARS-CoV-2 variants. We conducted antiviral experiments on the alpha and beta variants to verify the effectiveness of angeloylgomisin O and schisandrin B against the SARS-CoV-2 variants. 

**Figure 8:**
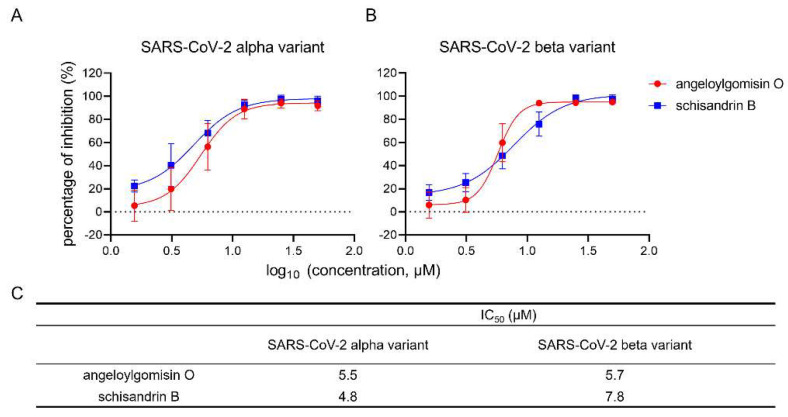
Antiviral activity of angeloylgomisin O and schisandrin B against the SARS-CoV-2 variants. (**A**,**B**) Dose-dependent curves of both angeloylgomisin O and schisandrin B against the alpha variant (**A**) and beta variant (**B**). (**C**) The IC_50_ values. Caco-2 cells were seeded in 96-well plates. After overnight incubation, cell monolayers were treated with hits; 1 h later, cells were infected with SARS-CoV-2 variants at an MOI of 0.5, followed by incubation for 24 h. The inhibition of SARS-CoV-2 variants was measured by using the IFA assay. Data were presented as the means ± SD from two or three independent experiments.

The results showed that angeloylgomisin O and schisandrin B inhibited the SARS-CoV-2 variants in a dose-dependent manner (Figure 8A,B). Angeloylgomisin O and schisandrin B showed similar IC_50_ values against the variants of concern and wild-type strains (Figure 8C). This result suggests that angeloylgomisin O and schisandrin B act as broad-spectrum antiviral agents against the emerging SARS-CoV-2 variants of concern.

## 4. Discussion

In this study, we screened a botanical drug library containing 1037 compounds and identified four hits, including angeloylgomisin O, procyanidin, schisandrin B, and oleanonic acid, which blocked the entry of SARS-CoV-2 S (ct19) pv by inhibiting viral membrane fusion. All four hits inhibited MERS-CoV S pv, SARS-CoV-2 S D614G pv, and SARS-CoV-2 S pv K417N/E484K/N501Y/D614G infection. Furthermore, the four hits inhibited the SARS-CoV-2 authentic virus at the micromolar level. The top two compounds, angeloylgomisin O and schisandrin B, inhibited authentic SARS-CoV-2 and SARS-CoV-2 alpha and beta variants at a highly selective index (SI). The combination of remdesivir-angeloylgomisin O showed more potent antiviral activity against SARS-CoV-2 than remdesivir monotherapy. 

The top two hits, angeloylgomisin O and schisandrin B, were derived from Schisandra chinensis. Fruits from this plant and its extracts have been used in traditional medicines in East Asia to treat liver disorders such as hepatitis. They also possess a broad spectrum of biological and pharmacological uses, including antiviral, anti-inflammatory, and antioxidative properties, without toxicity [36]. Schisandrin B has been shown to inhibit cytochrome P450 3A (CYP3A) activity in the rat liver, thus affecting the metabolism of many drugs [37,38]. Furthermore, upon oral administration of Schisandra chinensis extract in rats, schisandrin B accumulates to a maximum plasma concentration (C_max_) of 4.54 μM [39]. This value was similar to the IC_50_ value for schisandrin B in the present study, suggesting that this compound offers a biologically feasible treatment for SARS-CoV-2.

CD4+T cells are rapidly activated at the cellular level to produce inflammatory cytokines after SARS-CoV-2 infection, which further induces CD14+ CD16+ monocyte activation with high levels of interleukin 6 (IL-6) expression. Thus, reduced IL-6 could potentially reduce the immunopathology of SARS-CoV-2 [40,41]. Cai et al. demonstrated that a pretreatment of 8–10-week-old BALB/c mice with schisandrin B at doses of 25, 50, and 75 mg/kg reduced lipopolysaccharide-induced acute lung injury by lowering the number of inflammatory cells and proinflammatory cytokines, including tumor necrosis IL-6 in bronchoalveolar lavage fluid [42]. This result suggested that schisandrin B could coordinate the cytokine response to reduce the host immune response, and schisandrin B might achieve a better therapeutic effect in vivo.

Procyanidin is a pro-anthocyanidin member that belongs to the group of flavonoids, the secondary metabolites of polyphenolic plants and fungi. Polyphenolic compounds, such as flavonoids, are characterized by different biological activities, including antimicrobial, anticancer, anti-inflammatory, and antiviral properties [43]. Pro-anthocyanidins are also the most abundant polyphenolic compounds in lignin [16]. Maroli et al. used molecular docking analyses to demonstrate that procyanidin might inhibit SARS-CoV-2 entry and replication [44]. Our study demonstrated that procyanidin has virucidal and antiviral activity with a micromolar range.

Viral variants of concern may emerge with dangerous resistance to the immunity generated by the current vaccines to prevent COVID-19. For example, the alpha and beta variants are of concern because of their rapid rise to dominance and their extensive spike mutations, which may be detrimental to antiviral effectiveness and vaccine protection. Our results confirmed the broad-spectrum antiviral activity of angeloylgomisin O and schisandrin B against both variants. This result enhances the potential of angeloylgomisin O and schisandrin B as therapeutic agents for COVID-19.

## Data Availability

Not applicable.
